# A review of human sensory dynamics for application to models of driver steering and speed control

**DOI:** 10.1007/s00422-016-0682-x

**Published:** 2016-04-16

**Authors:** Christopher J. Nash, David J. Cole, Robert S. Bigler

**Affiliations:** Cambridge University Engineering Department, Trumpington Street, Cambridge, CB2 1PZ UK

**Keywords:** Sensory dynamics, Driver modelling, Perception thresholds, Sensory integration, Driver–vehicle dynamics

## Abstract

In comparison with the high level of knowledge about vehicle dynamics which exists nowadays, the role of the driver in the driver–vehicle system is still relatively poorly understood. A large variety of driver models exist for various applications; however, few of them take account of the driver’s sensory dynamics, and those that do are limited in their scope and accuracy. A review of the literature has been carried out to consolidate information from previous studies which may be useful when incorporating human sensory systems into the design of a driver model. This includes information on sensory dynamics, delays, thresholds and integration of multiple sensory stimuli. This review should provide a basis for further study into sensory perception during driving.

## Introduction

The continued development of advanced driver assistance systems (ADAS) in road vehicles is resulting in increasingly complex interactions between driver and vehicle (Gordon and Lidberg 2015). However, the role of the human driver in controlling the vehicle is still poorly understood. Consequently the vehicle development process still relies heavily on subjective evaluation of prototype vehicles by test drivers, which is expensive and time consuming. By building a deeper understanding of the interactions between driver and vehicle, models can be developed to assist with the design and evaluation of vehicle components and systems. One feature of driver–vehicle control that has been neglected to date is the sensory perception of the driver. The aim of this paper is to review the role of human sensory systems in the driving task, with a view to improving the capability of mathematical models of the driver.

Driving a vehicle involves a wide range of information processing levels, from the high-level navigation task to the low-level control of vehicle speed and direction. The focus of this review is on the role of human sensory dynamics in the low-level control task. Donges (1978) considered the steering control task as the superposition of a target following task (feedforward control) and a disturbance rejection task (feedback control). Disturbances may act on the vehicle from sources such as wind gusts, uneven road surfaces and nonlinearities in the vehicle dynamics, or they may originate from the driver due to physiological noise sources, constraints and nonlinearities.

**Fig. 1:**
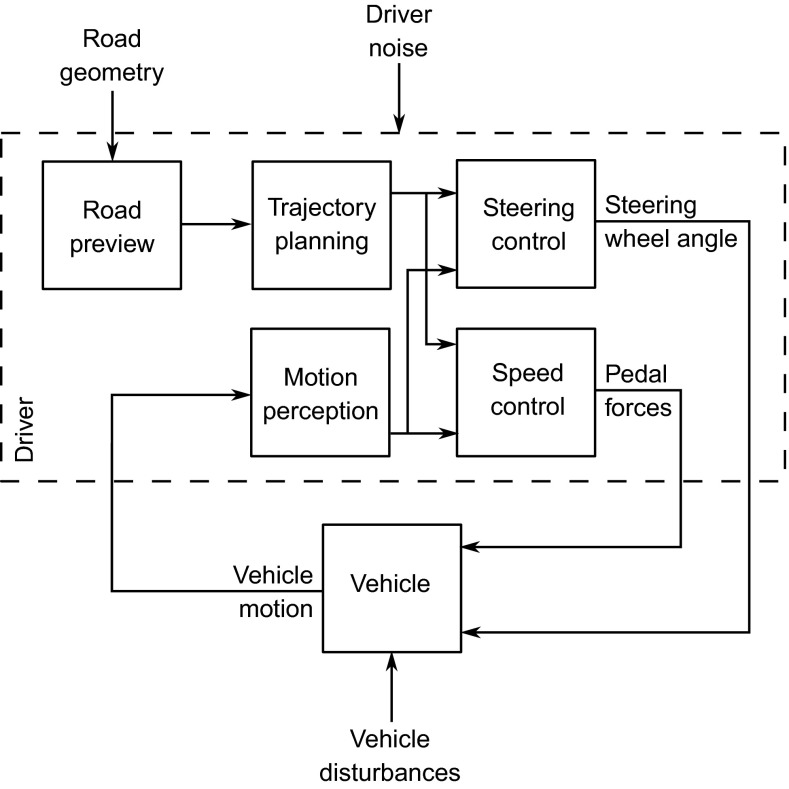
Block diagram of tasks carried out while driving. The driver must use their perceived information about the motion of the vehicle and the upcoming road geometry to plan a desired trajectory and then calculate the required steering wheel angle and pedal forces to achieve this trajectory as closely as possible

A simplified block diagram of the feedforward and feedback control of vehicle direction and speed is shown in Fig. 1. The driver previews the future road path using their visual system and then, using an internal model of the vehicle dynamics, determines target path and speed profiles and corresponding feedforward control actions (Timings and Cole 2013, 2014). Simultaneously, the driver senses the motion of the vehicle in relation to the target profiles and generates feedback control actions to reduce the effect of disturbances. The hypothesis presented in Fig. 1 assumes that feedback of vehicle motion is not used directly for generating the feedforward control action; however, the feedback loop is able to correct for any discrepancies introduced by imperfections in the driver’s feedforward control. It has been found that without visual feedback during lane change or obstacle avoidance manoeuvres drivers do not always initiate the return phase of the manoeuvre, failing to steer back towards the target path (Wallis et al. 2002; Cloete and Wallis 2009).

**Fig. 2:**
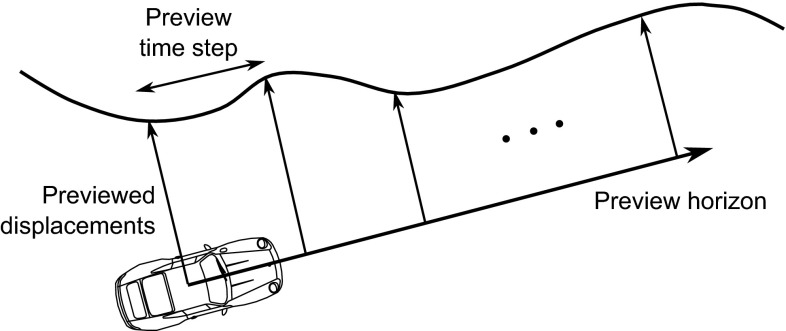
‘Preview’ model of drivers’ perception of the upcoming road path, used for feedforward steering and speed control (Sharp and Valtetsiotis 2001). The driver looks straight ahead and takes a series of measurements of the lateral displacement of the target path up to the ‘preview horizon’

Modelling the driver mathematically has been the subject of research for many decades. Comprehensive reviews are provided by Macadam (2003) and Plöchl and Edelmann (2007). Recent research has focussed on the application of optimal control theory, using model predictive or linear quadratic controllers that are able to preview the future road path, as shown in Fig. 2, and calculate an optimal sequence of control actions (Macadam 1981; Sharp and Valtetsiotis 2001; Peng 2002; Cole et al. 2006). This approach has been extended to include neuromuscular dynamics (Pick and Cole 2007, 2008; Odhams and Cole 2009; Abbink et al. 2011; Cole 2012) and to the control of nonlinear vehicle dynamics (Ungoren and Peng 2005; Thommyppillai et al. 2009; Keen and Cole 2011). Feedforward and feedback control are usually assumed to share a common objective function. Timings and Cole (2014) synthesised independent feedforward and feedback controllers to examine in more detail the robustness of the driver’s control strategy to disturbances.

While driver steering control has a fairly well-defined objective, to follow a target line and stay within road boundaries, the motivation for driver’s speed choice depends on the situation. In a normal driving situation drivers will balance factors such as safety, comfort, journey time and control effort (Prokop 2001; Odhams and Cole 2004). Drivers have been found to decrease their speed to minimise their lateral acceleration in corners (Ritchie et al. 1968; Herrin and Neuhardt 1974; Reymond et al. 2001). Road width has also been found to affect speed choice, with drivers adjusting their speed to remain within lane boundaries (Bottoms 1983; Defazio et al. 1992). In contrast, racing drivers aim to maximise their lateral acceleration within the limits of the tyres in order to minimise lap time (Timings and Cole 2014; Lot and Dal Bianco 2015). In situations with heavy traffic, driver speed choice may also be dictated by the speed of other vehicles, with the driver aiming to maintain a safe distance behind the car in front (Boer 1999; Kondoh et al. 2008).

Despite these developments, most models assume the driver has full knowledge of the vehicle states, and no existing driver models appear to take full advantage of current understanding of human sensory dynamics. While this review is primarily focussed on driving of road vehicles, clear parallels can be drawn with research into pilots in the aerospace industry. Indeed, sensory dynamics have been considered in greater detail in this area, and many of the studies cited in this review have come from work carried out by aerospace engineers to investigate human perception during control tasks. In particular, models of sensory dynamics have been used in studies carried out in flight simulators to understand how sensory information is used during real and simulated flight (Pool et al. 2008; Ellerbroek et al. 2008; Nieuwenhuizen et al. 2013; Drop et al. 2013; Zaal et al. 2009a, c, 2010, 2012, 2013).

**Fig. 3:**
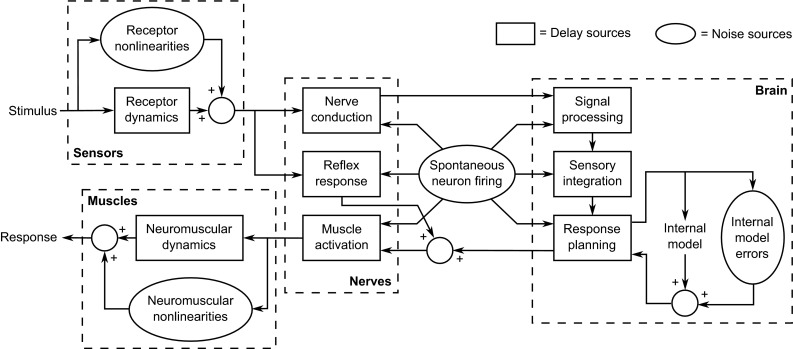
Diagram of the main processes carried out by the sensorimotor system to generate a physical response to a sensory stimulus. Stimuli are perceived by various sensors, which transmit electrical signals through the nerves to the brain. The brain processes and integrates these sensory signals and then plans a response using an internal model of the environment. The planned signals are sent to the muscles and shaped by the neuromuscular dynamics to give a physical response. There are various sources of time delays, shown by *boxes*, and noise, shown by *ovals*

Driving is just one of many human sensorimotor tasks that involve perceiving stimuli in the surrounding environment and responding with a physical action. The neurophysiological processes involved in such tasks are shown in Fig. 3. A stimulus may excite various senses, which produce chemical signals characterised by the dynamics of the sensory receptors (explored in Sect. 2). Sensory signals are then transmitted through the nerves as electrical impulses caused by firing neurons, with the firing rate encoding a frequency-modulated signal (Carpenter 1984). Certain stimuli can elicit reflexive responses which bypass the brain by activating motor neurons emerging from the spinal cord (Carpenter 1984).

There are physical and biochemical limitations to the speed with which each of the processes shown in boxes in Fig. 3 can be carried out; therefore, time delays are introduced into the sensorimotor system. These delays are discussed further in Sect. 3. In addition, noise is introduced due to nonlinearities in the receptor and neuromuscular dynamics, errors in the brain’s internal models and spontaneous firing of neurons (Fernandez and Goldberg 1971). This means that humans are unable to measure stimuli with perfect accuracy or plan and execute an ideal response. It also results in thresholds below which stimuli cannot be perceived, as discussed in Sect. 4.

Once the sensory signals are received in the brain, they are processed in the sensory cortex in order to extract the information from the encoded signals transmitted through the nerves (Kandel et al. 2000). The information from the different senses is then integrated to form a single representation of the surrounding environment, as explained further in Sect. 5. Based on this, the physical response to the perceived stimuli is planned using internal models of the human body and the surrounding world (Wolpert and Ghahramani 2000). The signals required to activate the muscles are generated in the motor cortex and fine-tuned in the cerebellum using feedback from the sensory measurements (Kandel et al. 2000). Signals are then transmitted along motor neurons which activate muscle fibres, causing them to contract. The physical response is shaped by the dynamic properties of the activated muscles. In the context of driving, earlier studies have measured and modelled the neuromuscular dynamics of drivers’ arms holding a steering wheel (Pick and Cole 2007, 2008; Odhams and Cole 2009; Cole 2012) and legs actuating a gas pedal (Abbink et al. 2011).

An important feature of perception during driving tasks is that the stimuli perceived by the driver’s sensory systems arise from the motion of the vehicle, which is controlled by the driver. This means that the driver is involved in an *active* closed-loop perception and control task, as opposed to a passenger who is a *passive* observer (Flach 1990). The driver is able to anticipate future motion of the vehicle, allowing more accurate sensory integration as discussed in Sect. 5. Driving also involves many sensory stimuli being presented simultaneously in different axes and stimulating different sensors (multimodal) compared with sensory measurements which have been carried out in one axis to stimulate one sensor (unimodal). Care must be taken when relating results from investigations carried out in passive, unimodal conditions to models of active, multimodal control and perception. This is discussed in relation to time delays in Sect. 3 and sensory thresholds in Sect. 4.

The scope of this review is broad, and thus it is not possible to review every topic in great detail; each section could be extended significantly. However, the aim of the review is to give an overview of the key results from the literature, with particular focus on motivating and informing further development of driver models incorporating human sensory system dynamics. Both steering and speed control are considered concurrently, since in many cases the sensory mechanisms discussed are relevant for both control tasks. The main findings of the review are summarised and discussed in Sect. 6. The review extends considerably an earlier review by Bigler and Cole (2011).

## Sensory dynamics

Various sensory systems are used by the driver to infer the state of the vehicle and its surroundings. The main sensory systems used in the control of vehicle speed and direction are:

- *Visual:* The visual system is the only means the driver has of detecting the upcoming road geometry. The visual system can also sense the motion of the vehicle relative to the surrounding environment.
- *Vestibular:* The vestibular organs are located within the inner ear, and they sense rotations and translations of the driver’s head.
- *Somatosensory:* Somatosensors include a wide range of sensory organs which detect various states of the body, such as contact pressure, temperature, limb position and pain. They include proprioceptors which detect joint angles, muscle lengths and tensions and their derivatives.

The following subsections give an overview of the published literature on these three sensory systems. Other senses such as hearing may also play a role but will not be discussed in detail.

### Visual system

Visual perception is the subject of significant research activity in psychology, neuroscience and biology. There is still much to understand about how a human interprets the neural signals received by the retina from a potentially complex three-dimensional visual scene containing objects that might be familiar or unfamiliar, and moving or stationary, with a moving or stationary observer. The various processes involved in visual perception are discussed in detail by Gibson (1950), Johansson (1975), Ullman (1979), Nakayama (1985), Lappe et al. (1999) and Raudies and Neumann (2012). Human visual perception is a complex, multi-layered process, and for the purpose of driver modelling it is not necessary or feasible to model all aspects. Therefore, the focus of this review is on the most relevant results towards modelling visual perception in a driving environment.

In the two-level model of vehicle control (Donges 1978), the visual system is used in both the feedback task and the feedforward task. The feedback task involves using the visual system in combination with the vestibular and somatosensory systems to perceive the motion of the driver and thus of the vehicle, which in turn is used to perform feedback control of the vehicle. In the feedforward task, the visual system views the geometry of the road ahead of the vehicle so that feedforward control inputs to the vehicle can be generated. Higher levels of the driving task, not considered in this review, involve the visual system in perceiving additional information such as motion of other vehicles and pedestrians.

**Fig. 4:**
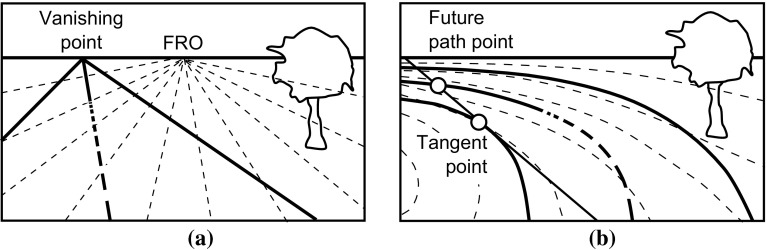
Potential candidates for visual cues used while driving along **a** a straight road and **b** a curved road. Optic flow patterns are shown by *dashed lines*. Drivers have also been found to use other objects and the road boundaries and centre line as visual references. Points such as the ‘tangent point’ and ‘future path point’ have been identified as fixation points for feedforward visual perception.

#### Perception of self-motion (feedback)

Visually induced motion perception is typically caused by motion of the eyes relative to fixed surroundings, although illusory self-motion perception known as vection can also be induced by moving surroundings (Dichgans and Brandt 1978). Since vehicle motion is primarily planar, the role of the driver’s visual system in perceiving self-motion is mainly concerned with three axes: longitudinal and lateral translations, and yaw (heading) rotations.

Various mechanisms have been suggested for visual motion perception, such as ‘optic flow’ (Gibson 1950; Koenderink 1986; Lappe et al. 1999). This is the velocity field created as points in the visual scene ‘flow’ over the retina, along lines known as streamers. Optic flow patterns while driving on straight and curved roads are shown by the dashed lines in Fig. 4. For straight motion, the streamers all originate from a point directly in front of the observer known as the ‘focus of radial outflow’ (FRO). This can be used as a visual cue to control the vehicle’s heading direction (Gibson 1950), for example by aligning with the ‘vanishing point’ at the end of a straight road. For rotational motion, the streamers are curved and the FRO does not exist, although the point on the horizon directly in front of the observer may still be used as a visual cue to heading direction (Grunwald and Merhav 1976). However, Riemersma (1981) suggested that the FRO and heading direction are too crude to play a role in car driving. Multi-level models of perception of motion from optic flow have been developed (Grossberg et al. 2001; Mingolla 2003; Browning et al. 2009); however, these descriptions do not lead easily to a simple relationship between vehicle motion and visually perceived motion, as they are dependent on the characteristics of the surroundings.

Alternatively, it has been proposed that humans measure the rates of change of vectors between themselves and specific objects in the visual field (Gordon 1965; Zacharias et al. 1985). This allows drivers to calculate their ‘time-to-collision’ with objects, which can be particularly useful when following a leading vehicle (Kondoh et al. 2008). The distance and relative velocity of the objects can only be inferred with prior knowledge of the object’s size or by comparison between two visually similar environments (Gordon 1965; Bremmer and Lappe 1999). Road edges and centre line have also been identified as key visual features used by drivers (Gordon 1966; Riemersma 1981).

Because of the variety of mechanisms involved in visual perception, it is difficult to say what constitutes the ‘input’ to the visual system. Optic flow models would suggest that velocities are measured, although the FRO can be used to measure heading direction (yaw angle), and it is clearly possible to discriminate translational displacements with reference to stationary features such as road markers. Gordon (1965) used the unnatural appearance of the acceleration field to argue that accelerations and higher derivatives are not directly sensed by the visual system. The most appropriate inputs to the feedback component of the driver’s visual system therefore appear to be translational and angular velocities. Since displacements and angles can only be measured with respect to references such as road markers, they can be included within models of drivers’ feedforward visual perception.

It is not clear from the mechanisms involved in visual perception whether the perceived rotational and translational velocities depend on the frequency of the stimulus. One possible approach is simply to assume unity gains between the actual and perceived velocities. An alternative estimate of the frequency response of the visual system may be obtained from sensory threshold measurements (Soyka et al. 2011, 2012, see Sect. 4 for more information). Riemersma (1981) and Bigler (2013) both measured thresholds of visual perception of lateral and yaw velocities, superimposed on a constant longitudinal velocity. Both studies presented subjects with a typical driving scene, with Riemersma (1981) displaying edge lines for a straight road and Bigler (2013) displaying a more realistic rendering of a straight road bordered by trees. Riemersma (1981) found that lateral and yaw thresholds were independent of longitudinal speed. Bigler (2013) found thresholds for stimuli of different frequencies, and reanalysing the results using the model of (Soyka et al. 2011, 2012) gives visual dynamics that can be described by a low-pass filter, given by:

1 $$\begin{aligned} H_{\mathrm{vi}}(s) = \dfrac{\omega _{\mathrm{vi}}}{s + \omega _{\mathrm{vi}}} \end{aligned}$$

and taking lateral velocity and yaw angular velocity as inputs. The same cutoff frequency $\omega _{\mathrm{vi}} = 0.810$ rad/s was found to fit the results for both sway and yaw motion. This low-pass characteristic was also seen by Riemersma (1981). In the absence of direct measurements of nervous responses to sensory stimulation, this model inferred from sensory threshold data can be used to give some insight into the function of the visual system. However, further research is needed to validate this approach.

#### Perception of road path geometry (feedforward)

One of the key characteristics of driving tasks is the ability of the driver to use their visual system to ‘preview’ the road ahead in order to carry out feedforward control. Studies have investigated the key features of road geometry which are perceived while driving, often using eye tracking instrumentation to investigate where the drivers look. Shinar et al. (1977) found a difference between straight roads, where drivers tend to focus near the FRO, and curved roads, where drivers scan the geometry of the curve. Many studies have found that drivers focus on the ‘tangent point’ on the inside of a bend, as shown in Fig. 4 (Land and Lee 1994; Boer 1996; Kandil et al. 2009, 2010). The angle between the current vehicle heading vector and the tangent point can be used to estimate the road curvature (Land and Lee 1994) and required steering angle (Kandil et al. 2009). Other studies have suggested drivers may look at a point on the predicted vehicle path, the ‘future path point’ (Land 1998) as shown in Fig. 4. There is no overwhelming evidence in favour of the tangent point over the future path point or other nearby points as a fixation point during driving (Mars 2008; Robertshaw and Wilkie 2008; Lappi et al. 2013).

Eye tracking studies have found that drivers tend to focus on a point around 1–2 s ahead of the vehicle on straight roads (Land and Lee 1994; Donges 1978), and that their gaze tends to move to an upcoming curve around 1 s before they steer in that direction (Chattington et al. 2007; Land and Tatler 2001). Drivers have also been found to make short ‘look-ahead fixations’, looking further along the road for short periods of time (Lehtonen et al. 2013). While eye tracking instrumentation is useful for determining the gaze direction of a driver, Land and Lee (1994) noted that it does not necessarily indicate where the driver is directing their attention, because the driver may be using their peripheral vision to gather information about road geometry away from the gaze point. Grunwald and Merhav (1976) and Land and Horwood (1995) both measured driver performance with only certain parts of the road visible and found that the full visual control task can be represented by two viewing points, one near to the driver and one further down the road. Land and Horwood (1995) found that performance was not degraded from the full visibility condition if drivers could see a near point 0.53 s ahead and a distant point 0.93 s ahead.

Steen et al. (2011) reviewed many studies which proposed one, two or multi-point preview models and concluded that a two-point preview model was the most realistic, with one point close to the driver and one more distant point. However, Sharp and Valtetsiotis (2001) used a shift register to formulate a multi-point preview controller using visual information taken from a single preview point, suggesting that a human driver in a moving vehicle could use memory to construct a multi-point image of the road geometry from data sensed at just one or two discrete points. The use of linear quadratic optimal control theory to calculate the gains on multi-point road path geometry ahead of the vehicle shows that the gains eventually tend to zero as the time ahead of the vehicle increases. This indicates that looking beyond a certain point might result in diminishing returns (Sharp and Valtetsiotis 2001; Cole et al. 2006), with the time ahead of the vehicle at which this occurs dependent on the dynamic properties of the vehicle and the driver, and the amount of control effort applied by the driver.

### Vestibular system

There is some disagreement in the literature as to the relative importance of the vestibular system in nonvisual motion perception. Studies measuring thresholds of human motion perception in the dark often assume that the influence of the vestibular system is much larger than that of the somatosensors (Benson et al. 1986, 1989; Grabherr et al. 2008; Soyka et al. 2012, 2009, 2011; Kingma 2005). However, Gianna et al. (1996) found that perception thresholds for subjects with vestibular deficiencies were not significantly higher than for normal subjects, and Bronstein and Hood (1986) found that neck proprioception largely replaced vestibular function in vestibular deficient subjects for head rotations relative to the body. In contrast, Mallery et al. (2010) found that a subject with vestibular deficiencies had rotational velocity thresholds an order of magnitude higher than those of normal subjects and Valko et al. (2012) found that vestibular deficient subjects had significantly higher perception thresholds in four different motion axes. The relative importance of the vestibular and somatosensory systems may depend on the precise nature of the stimuli; however, it does appear that the vestibular system is an important source of information for drivers.

The vestibular system consists of two sets of organs located in the inner ear: the semicircular canals (SCCs) which sense rotational motion and the otoliths which sense translational motion (Kandel et al. 2000). Many studies have investigated the function of the vestibular system in primates and humans, either directly by measuring electrical signals in the brain or indirectly by measuring the vestibulo-ocular reflex (VOR), a reflexive eye movement which uses vestibular information to compensate for head movements.

#### Otoliths

The otoliths are formed from small granular particles contained in a gelatinous membrane which is in turn connected to sensory cells via hairs called cilia. When subjected to translational acceleration, the inertia forces on the otoliths deflect the cilia and excite the sensory cells (Kandel et al. 2000). Most mathematical models are based on empirical data from experiments carried out on humans and animals.

It is a natural extension of Einstein’s equivalence principal (Einstein 1907) that humans cannot tell the difference between a translational acceleration and a change in orientation of the gravity vector. Young and Meiry (1968) developed a model for the otoliths relating the perceived specific force (combination of inertial and gravitational accelerations) to the actual specific force. They proposed the transfer function:

2 $$\begin{aligned} H_{\mathrm{oto}}(s) = K_{\mathrm{oto}} \left[ \dfrac{(1+T_{\mathrm{oto1}} s)}{(1+T_{\mathrm{oto2}} s)(1+T_{\mathrm{oto3}} s)}\right] \end{aligned}$$

and identified values for its parameters, given in the first row of Table 1. With these values, the transfer function is essentially low pass but with a constant reduction in gain at very low frequencies.

**Fig. 5:**
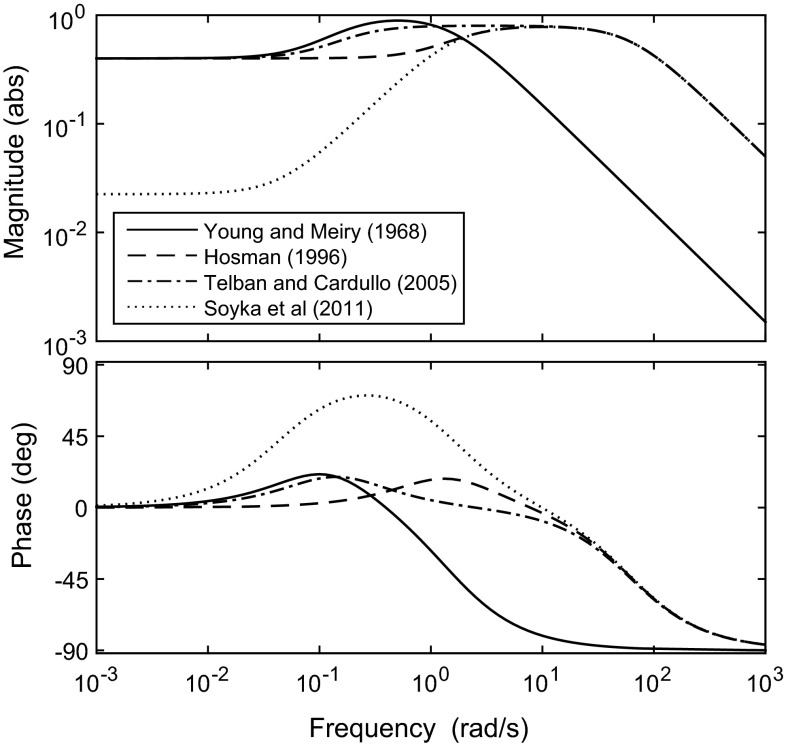
Bode plot for otolith transfer function with parameters from different studies, given in Table 1. Input is acceleration

Fernandez and Goldberg (1976) measured the afferent firing rate (AFR) in the brains of squirrel monkeys subjected to accelerations at various frequencies and magnitudes. They developed a model of the otoliths containing a fractional exponent, which is difficult to implement practically. Therefore, Hosman (1996) proposed a simplified version in the same form as Eq. 2. Based on this and other research, Telban and Cardullo (2005) suggested parameters for a transfer function in the form of Eq. 2, relating the specific force input to a perceived specific force output. Soyka et al. (2011) used a signal-in-noise model to find a transfer function for the otoliths which optimised the fit to sensory threshold measurements (see Sect. 4 for more information). Suggested otolith parameters from these studies are summarised in the remaining rows of Table 1. The gains $K_\mathrm{oto}$ have been adjusted to give comparable outputs, since the scaling of the output signal is arbitrary. Bode plots of the otolith transfer function using the different parameters are compared in Fig. 5. For a driving task, the mid-range frequencies (between around $10^{-1}$ and $10^{1}$ rad/s) are the most important, and in this range the otoliths exhibit a roughly proportional response to accelerations. There are differences in the details of the frequency responses measured in different studies, which highlights the difficulty in achieving repeatable results when using different subjects, equipment and methodologies.

**Table 1:** Otolith model parameters

Study	$K_{\mathrm{oto}}$	$T_{\mathrm{oto1}}$	$T_{\mathrm{oto2}}$	$T_{\mathrm{oto3}}$
Young and Meiry (1968)	0.4	13.2	5.33	0.66
Hosman (1996)	0.4	1	0.5	0.016
Telban and Cardullo (2005)	0.4	10	5	0.016
Soyka et al. (2011)	0.0225	22.05	0.62	0.016

#### Semicircular canals

The semicircular canals consist of sets of three elliptical cavities which are each filled with fluid (Kandel et al. 2000). Angular motion about any axis causes the fluid to move within these cavities, causing deflections of small hair cells which excite sensory cells. Early models of the SCCs were based on considerations of the physical dynamics of the organs. Steinhausen (1933) used observations of the motion within the SCCs of fish to develop the ‘torsion-pendulum’ model. Young and Oman (1969) adapted this model to include additional ‘adaptation’ terms $T_{\mathrm{SCCa}}$ to match trends seen in experimental results. Fernandez and Goldberg (1971) added a lead term $T_{\mathrm{SCC1}}$, giving the transfer function:

3 $$\begin{aligned} H_{{\mathrm{SCC}}}(s) = K_{\mathrm{SCC}}\left[ \dfrac{T_{\mathrm{SCCa}} s}{(1+T_{\mathrm{SCCa}} s)}\right] \left[ \dfrac{(1+T_{\mathrm{SCC1}}s)}{(1+T_{\mathrm{SCC2}} s)(1+T_{\mathrm{SCC3}} s)}\right] \nonumber \\ \end{aligned}$$

which relates the AFR to the angular acceleration of the stimulus.

**Table 2:** SCC model parameters

Study	$K_{\mathrm{SCC}}$	$T_{\mathrm{SCCa}}$	$T_{\mathrm{SCC1}}$	$T_{\mathrm{SCC2}}$	$T_{\mathrm{SCC3}}$
Fernandez and Goldberg (1971)	5.73	80	0.049	5.70	0.005
Hosman (1996)	5.73	(80)	0.110	5.90	0.005
Telban and Cardullo (2005)	5.73	80	(0.060)	5.73	(0.005)
Soyka et al. (2012)	2.2	($\infty$)	0.014	2.16	0.005

Parameters which the authors have suggested may be neglected are given in brackets

**Fig. 6:**
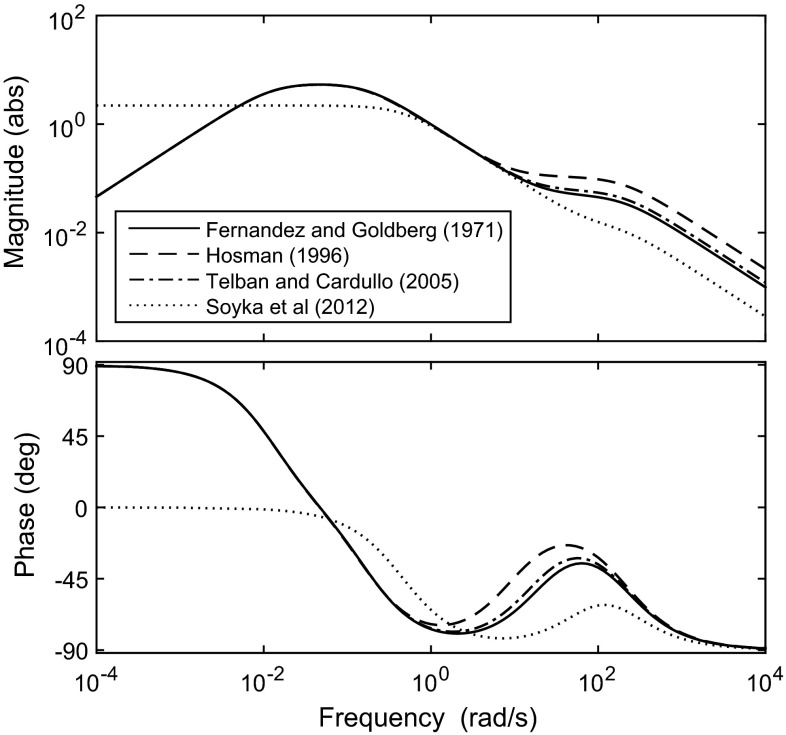
Bode plot for SCC transfer function with parameters from different studies, given in Table 2. Input is angular acceleration

Fernandez and Goldberg (1971) measured the AFR of squirrel monkeys in response to angular accelerations of various amplitudes and frequencies. Hosman (1996) suggested alternative parameter values based on results from the literature, neglecting the adaptation time constant $T_{\mathrm{SCCa}}$ since it lies outside the bandwidth of interest for driving tasks. Telban and Cardullo (2005) reviewed several relevant studies and suggested slight modifications to the parameters of Eq. 3. They also proposed a simplified transfer function for modelling purposes, which links angular *velocity* inputs (hence the $s^2$ term) to perceived angular velocity outputs:

4 $$\begin{aligned} H_{{\mathrm{SCC}}}(s) = K_{\mathrm{SCC}}\left[ \dfrac{T_{\mathrm{SCCa}} s^2}{(1+T_{\mathrm{SCCa}} s)(1+T_{\mathrm{SCC2}} s)}\right] \end{aligned}$$

(there is a typographical error in (Telban and Cardullo 2005), with *s* in the numerator instead of $s^2$). This transfer function neglects the short time constants $T_{\mathrm{SCC1}}$ and $T_{\mathrm{SCC3}}$, which affect frequencies well above the range of normal head movements. The key feature of the transfer function is roll-off below about $10^{-2} \hbox { rad/s}$ which means that there is zero response at constant angular acceleration. In the same way as for the otoliths (Soyka et al. 2011), Soyka et al. (2012) chose time constants to optimise the fit to sensory threshold measurements using a signal-in-noise model. Similarly to Hosman (1996), they neglected the adaptation time constant $T_{\mathrm{SCCa}}$. SCC parameters found from various studies are summarised in Table 2. As with the otoliths, the gains $K_\mathrm{SCC}$ have been adjusted to give comparable outputs. Bode plots of the SCC transfer function using the different parameters are compared in Fig. 6. At mid-range frequencies the transfer functions have the characteristics of an integrator, hence why Telban and Cardullo (2005) suggested the SCCs measure angular velocity rather than acceleration. In contrast to the otolith dynamics, the agreement between the different studies is much higher. This may be because these studies based their work on similar models of the physical dynamics of the SCCs, although the transfer function found from sensory thresholds (Soyka et al. 2012) also agrees well with the others at mid-range frequencies.

### Somatosensors

During driving, the information provided by the visual and vestibular systems is complemented by the response of various receptors of the somatosensory system (Kandel et al. 2000). A particular group of receptors provide proprioception, which is the sensing of joint angles and movements and muscle displacements and forces. These receptors are particularly important in allowing the driver to sense the angle and torque of the steering wheel, which can be used by experienced drivers to sense the characteristics of the contact between the tyre and the road. Proprioceptors are also used to sense the displacements and forces of the foot pedals. The following subsections discuss the properties of the muscle spindles, which measure muscle displacement, and the Golgi tendon organs, which measure muscle force. Other somatosensors which may play a role are skin receptors and joint receptors which give information on touch and joint angle (Collins et al. 2005; Proske and Gandevia 2009), and graviceptors which respond to the motion of fluid within the body (Vaitl et al. 2002). While these somatosensors may give the driver useful information, such as the contact forces between the body and the seat, the nature of these stimuli means they are difficult to measure and quantify, and as such the existing literature does not lend itself to application within driver models.

#### Muscle spindles

Muscle spindles are sensors which detect the length and rate of change of length of the muscles. They produce two separate signals, one dependent on muscle velocity and length (type Ia afferent) and one dependent on muscle length only (type II afferent) (Kandel et al. 2000). An empirical linear model of the muscle spindle response, based on measurements taken in cats, was formulated by Poppele and Bowman (1970), with the Ia and II afferent responses to muscle displacements given by:

5 $$\begin{aligned} H_{\mathrm{Ia}}(s)&= \frac{s(s+0.44)(s+11.3)(s+44)}{(s+0.04)(s+0.816)} \end{aligned}$$

6 $$\begin{aligned} H_{\mathrm{II}}(s)&= \frac{(s+0.44)(s+11.3)}{(s+0.816)} \end{aligned}$$

More complicated nonlinear models have also been developed which can predict the afferent responses accurately under a wide variety of conditions (Maltenfort and Burke 2003; Mileusnic et al. 2006).

#### Golgi tendon organs

Golgi tendon organs (GTOs) respond to the forces in the muscles. They share a nerve with the Ia afferent response of the muscle spindles, giving a response known as a type Ib afferent (Kandel et al. 2000). A linear model of the GTOs was first proposed by Houck and Simon (1967), again based on measurements in cats. Their model was stated as a transfer function between muscle force and Ib afferent response by Prochazka (1999):

7 $$\begin{aligned} H_{\mathrm{Ib}}(s) = 333\frac{(s+0.15)(s+1.5)(s+16)}{(s+0.2)(s+2)(s+37)} \end{aligned}$$

A nonlinear model of the GTOs has also been developed (Mileusnic and Loeb 2006) and has been found to describe the static and dynamic properties of the GTOs accurately.

## Time delays

As shown in Fig. 3, there are various ways in which delays are introduced between sensory stimuli being applied to a driver and the driver’s control response being measured. Delay sources include receptor dynamics, nerve conduction, neural processing and neuromuscular dynamics. Various techniques have been used in the literature to measure delays in human response to sensory stimulation. The simplest of these is to apply a stimulus and measure the time taken for a physical response (such as pressing a button) to be recorded. Some studies have used more sophisticated methods of applying stimuli, such as galvanic vestibular stimulation (GVS) which bypasses the vestibular organs by applying an electrical stimulus directly to the nerves (Fitzpatrick and Day 2004). Other methods have been used to detect responses at other points in the process, such as measuring the VOR to identify the reflexive delay, using magnetoencephalography (MEG, Hämäläinen et al. 1993) or electroencephalography (EEG) to measure electrical impulses within the brain or using electromyography (EMG) to record electrical activity in the muscles.

When interpreting sensory time delays measured in different studies using different techniques, it is important to consider which of the delay components shown in Fig. 3 are included in the measurement in each case. The aim of this section is to use results from the literature to estimate the total delay between stimulus and response for each sensory system. However, it can be difficult to separate the effects of pure time delays from lags due to the dynamics of the sensors and muscles and the time taken for signals to rise above noise levels (Soyka et al. 2013). Nevertheless, results from the literature can be used to find an approximate estimate of the order of magnitude of time delays in human sensory systems.

EMG has been used to measure the response of the muscle spindles to applied muscle stretches, finding delays of 25–30 ms for the Ia afferent and 40 ms for the II afferent (Matthews 1984). Bigler (2013) combined these with measured nerve conduction delays (Trojaborg and Sindrup 1969; Kandel et al. 2000) to give delays of 34 ms and 48 ms for the Ia and II afferents. As the Ib afferent response of the GTOs shares the same nerve as the Ia muscle spindle response, the time delay for the Ib afferent may be the same as the Ia muscle spindle response. However, these values do not include any neural processing time, so the actual sensor delays are likely to be larger.

Reaction times for drivers’ responses to simulated wind gusts have been measured in a driving simulator (Wierwille et al. 1983). Mean delays of 0.56 s without motion feedback and 0.44 s with motion feedback were found. These measurements encompass the complete process between stimulus application and physical response shown in Fig. 3, including all delays, lags and noise. Therefore, they can be considered as upper bounds for the delays in the visual system and combined visual–vestibular systems during driving. MEG has been used to record neural responses to visual stimuli and delays of 140–190 ms have been found (Kawakami et al. 2002; Lam et al. 2000), although it is unclear how much neural processing is carried out before and after this response is measured. Vestibular reflex delays have been measured by actively stimulating vestibular nerves using GVS and measuring the latency to the onset of the VOR (Aw et al. 2006; Tabak et al. 1997). Delays of 5–9 ms have been found, showing that the conduction of vestibular reflex signals is very fast.

There is a growing body of evidence, reviewed by Barnett-Cowan (2013) that despite the very fast conduction of vestibular reflex signals, vestibular processing can take much longer than the processing of other sensory signals. Vestibular delays have been found to be significantly longer than visual delays when measuring brain responses using EEG (Barnett-Cowan et al. 2010) and when measuring overall reaction times (Barnett-Cowan and Harris 2009). Barnett-Cowan et al. (2010) measured impulses in the brain 100 ms and 200 ms after visual and vestibular stimuli, respectively, with a further 135 ms until a button was pressed in both cases. This gives visual and vestibular delays of 235 ms and 335 ms; however, Barnett-Cowan (2013) suggested that these delays may include the time taken for the stimuli to rise above threshold levels (as modelled by Soyka et al. 2013) so they may be overestimates.

The visual and vestibular delays measured by Barnett-Cowan et al. (2010) are significantly lower than those found in a driving simulator by Wierwille et al. (1983). Furthermore, Barnett-Cowan et al. (2010) measured larger vestibular delays than visual delays, whereas Wierwille et al. (1983) found that adding vestibular stimuli significantly reduced the overall delay. This may indicate that sensory delays are dependent on the conditions in which the stimuli are applied. Delays due to nerve conduction and sensory and neuromuscular dynamics are a result of biochemical processes which are unlikely to depend significantly on the precise nature of the task carried out. However, it is likely that neural processing time is affected by the complexity of the task and the presence of distracting information and stimuli. Studies have investigated the intermittent nature of cognitive processing (Gawthrop et al. 2011; Johns and Cole 2015), which may play a part in increasing reaction times with increased mental load.

Rather than passively responding to stimuli as in many of these studies, drivers actively control the motion of the vehicle. It is difficult to measure time delays during an active control task, as response times are affected by the closed-loop dynamics. Some insight can be gained by looking at studies which have identified visual and vestibular delays during closed-loop pilot control tasks (Ellerbroek et al. 2008; Nieuwenhuizen et al. 2013; Zaal et al. 2009c, a, 2012, 2013). In general, vestibular delays have been found to be lower than visual delays, with vestibular delays between around 0.05–0.23 s and visual delays between around 0.18–0.32 s. These values seem consistent with the values measured in passive conditions; however, due to the large variability in measurements it is difficult to say whether delays are longer in active or passive conditions. Delays have been found to increase in the presence of additional stimuli (Zaal et al. 2009a) and in real flight compared with a simulator (Zaal et al. 2012). This indicates that perceptual delays are higher during multimodal conditions.

## Perception thresholds

Due to limits of human sensory organs and noise caused by spontaneous neuron firing, sensory systems have thresholds below which stimuli cannot be perceived. Perception thresholds are defined as the smallest stimulus which can be detected, and these are commonly measured by asking subjects to distinguish something about the stimulus, such as its direction. In reality, these thresholds are generally not precise, but a smooth transition from 0 to 100 $\%$ probability of detection over a range of values. This cumulative probability distribution is known as a ‘psychometric function’ (Boring 1917) and is often modelled as a cumulative normal distribution. Variations on the ‘up–down’ method (Levitt 1970) are commonly used to measure perception thresholds, and depending on the method used the thresholds measured correspond to different probabilities of detection, generally between 65 and 80 $\%$.

**Fig. 7:**
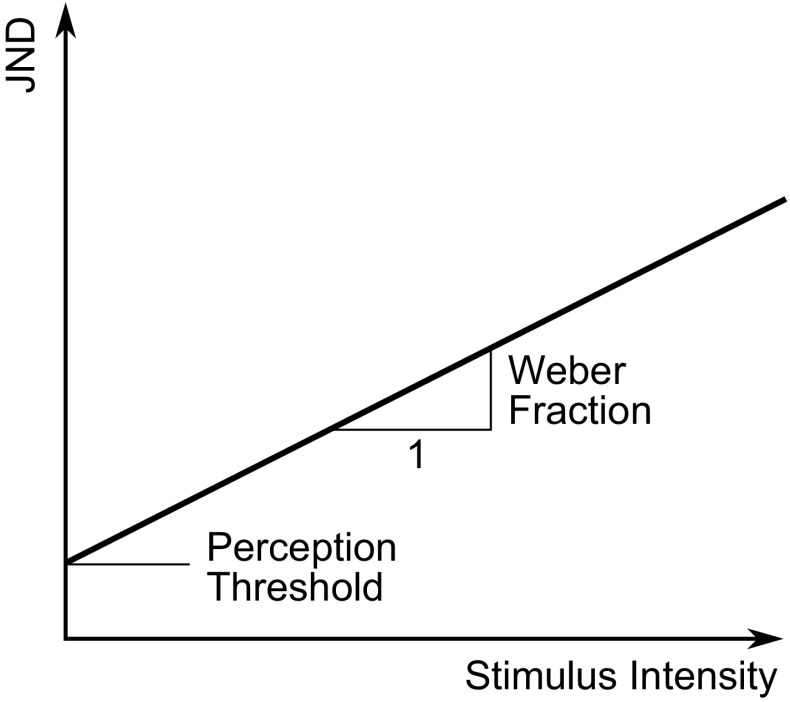
JNDs for a stimulus following Weber’s law. Weber’s law states that JNDs increase linearly with stimulus intensity. The constant of proportionality is known as the Weber fraction, and the y-intercept is the perception threshold

The ‘just noticeable difference’ (JND) is defined as the smallest change in amplitude from a reference stimulus which is required before the difference between the two stimuli is noticed. From experiments on the perception of lifted masses, Weber (1834) found that the JND in mass was proportional to the reference mass. This result has been found to be applicable for many perceptual systems and has become known as ‘Weber’s law’ with the constant of proportionality known as the ‘Weber fraction’. Figure 7 shows how the JND varies with stimulus intensity for a stimulus following Weber’s law.

Many of the published measurements of perception thresholds were carried out under passive, unimodal conditions, meaning that the test subjects were exposed only to the one stimulus of interest and they did not perform any task other than perceiving the stimulus. However, during driving multiple senses are being stimulated simultaneously in different axes, and the driver is carrying out an active control task. Groen et al. (2006) defined the ‘indifference threshold’ as the threshold for perception of a stimulus in the presence of other congruent or incongruent stimuli. JNDs are a special case of indifference thresholds, when the background stimulus is in the same axis and modality as the stimulus which is being detected. Another special case of the indifference threshold is for congruent stimuli from two different sensory modalities (e.g. visual and vestibular systems), where the indifference threshold marks out a ‘coherence zone’ of stimuli which are perceived as consistent with each other.

### Threshold models

**Fig. 8:**
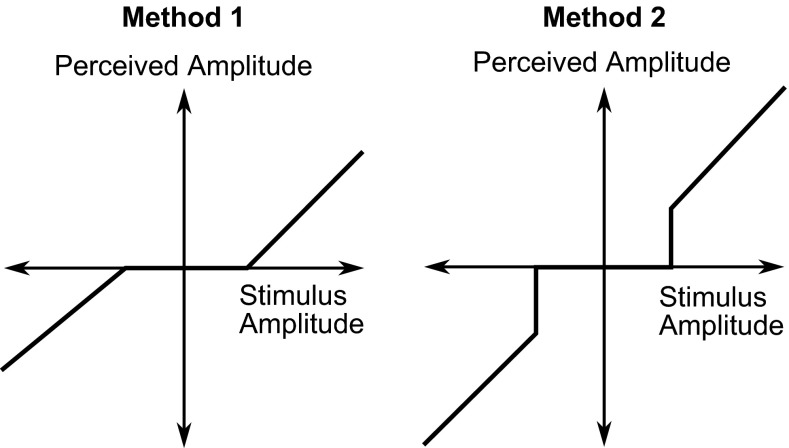
Two methods of modelling sensory thresholds as dead zones. In method 1, the perceived amplitude increases from zero after the threshold is reached, whereas in method 2 the perceived amplitude is equal to the stimulus amplitude above the threshold

The simplest model of sensory thresholds is a ‘dead zone’ where the perceived amplitude is zero. There are two possible methods for modelling this, as shown in Fig. 8. Method 2 is the most applicable of these, as method 1 implies that the perceived amplitude would be smaller than the actual amplitude, even above the perception threshold. The dead zone model is useful for simplicity; however, it assumes that the psychometric function is a step function, and it cannot be used directly to model JNDs.

**Fig. 9:**
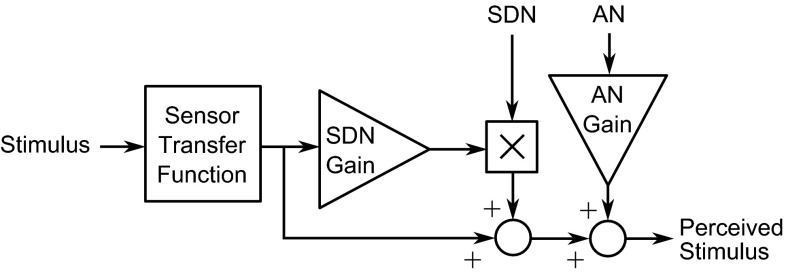
Sensor model incorporating additive and signal dependent noise (Bigler 2013). Noise is added after the sensor transfer function to represent spontaneous neuron firing in the brain. This is similar to the model of Soyka et al. (2011, 2012), who modelled thresholds using a constant noise addition after the sensory transfer function

Recent studies have suggested that sensory thresholds arise primarily as a result of noise in the sensory channels and the brain. Soyka et al. (2011, 2012) developed models of translational and rotational motion perception thresholds based on additive noise (AN) applied to the outputs of the otolith and SCC transfer functions. The perception thresholds were found as the minimum stimulus amplitude required for the output to exceed the noise level. Both studies found good fits to experimental results, although the transfer functions had to be adjusted slightly from those found in the literature (see Sect. 2). This model predicts the frequency dependence of perception thresholds and is valid for arbitrary motion inputs rather than solely sinusoidal motion. A similar principle was used by Bigler (2013) to model JNDs as well as perception thresholds, by adding signal-dependent noise (SDN) as well as AN to the output of the sensor transfer function (Todorov 2005). This sensor model is shown in Fig. 9.

### Passive threshold measurements

Thresholds and JNDs have been measured in passive conditions for a variety of stimuli. Soyka et al. (2011, 2012) showed that sensory thresholds could be predicted by finding when the output of the sensory transfer function rises above a specific noise amplitude; therefore, this model can be used in reverse to infer noise amplitudes from sensory threshold measurements. In the following subsections, noise amplitudes are found in this way for the different senses using sensory threshold measurements from the literature. These measurements have all been taken under passive unimodal conditions; therefore, since thresholds have been found to increase under active or multimodal conditions (see Sect. 4.3) the noise amplitudes found in this section can be considered to be lower bounds. For each sensory system, the signal-in-noise model of Soyka et al. (2011, 2012) has been used to identify the additive noise amplitudes using two different transfer functions: (i) a published sensor transfer function from considerations of the sensory dynamics and (ii) a sensor transfer function optimised to fit threshold data. It is unclear which of the two transfer functions is more appropriate for driver modelling. The parameters derived from sensory threshold measurements may describe the behaviour at low amplitudes better; however, they may not completely match the dynamic behaviour of the sensory system. Noise amplitudes are given in units with a * symbol at the end, to indicate that the noise is added to the stimuli filtered by the sensory transfer functions.

**Fig. 10:**
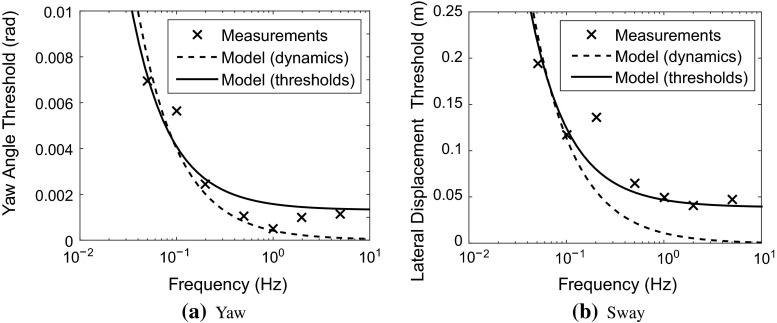
Visual feedback perception thresholds measured by Bigler (2013). Sinusoidal yaw angle and lateral displacement signals were superimposed on constant velocity forward motion and the minimum detectable motion measured. The visual display was a 2-dimensional screen which was not scaled to match the real-life motion amplitude, so the absolute values of the thresholds may not be at the correct scale. Model fits are shown using two different transfer functions, one from a simple model of the sensory dynamics of the visual system and one which was optimised to fit the measured thresholds

#### Visual thresholds

Various studies have measured perception thresholds and JNDs for the visual perception of self-motion. A difficulty in interpreting these results with any certainty is that they may well be dependent on the characteristics of the visual scene, such as the relative motion of stationary reference objects in the visual field, so it is not clear how generally applicable the results are. However, it may still be possible to find some useful information about the performance limits of the visual system.

A driving simulator display was used by Bigler (2013) to measure yaw angle and lateral displacement thresholds. The display was not calibrated to give full-scale visual feedback so the absolute values of the measured thresholds may not be at the correct scale; however, the frequency response should not depend on the display scaling. The results are shown in Fig. 10. The visual transfer function given in Eq. 1 was used with the model of Soyka et al. (2011, 2012) to give predicted thresholds, shown by the solid lines in Fig. 10. The model fits the thresholds very well, which is not surprising considering that the visual transfer function was found by fitting parameters to these results. The additive noise levels found are 0.0011 rad/s* for the yaw angular velocity and 0.032 m/s* for the lateral velocity.

A fit to the data was also found for a simple model of the visual system dynamics, with unity transfer functions between actual and perceived yaw and sway velocities. The fit using this model is shown by the dotted lines in Fig. 10, and the noise values found were 0.0013 rad/s* for the yaw angular velocity and 0.035 m/s* for the sway velocity. Visual JNDs have been measured for a range of yaw velocities, and Weber fractions of 7 % (de Bruyn and Orban 1988), 10 % (dos Santos Buinhas et al. 2013) and 11 % (Nesti et al. 2014b) have been found. No studies have been found which measure visual JNDs for lateral motion.

A few studies have investigated the limits of visual perception of motion in the longitudinal direction. Reinterpretation of the data collected by Bremmer and Lappe (1999) gives a JND in displacement in the longitudinal direction of 450 mm, with a reference displacement of 4 m. This gives a Weber fraction of 10 $\%$; however, extrapolating from measurements taken for this relatively short displacement of 4 m may be inaccurate. Monen and Brenner (1994) determined the smallest step increase in forward velocity necessary for the difference to be perceived within half a second and found a large Weber fraction of around 50 %.

Thresholds of visual perception involved in feedforward control have not been measured explicitly. Authié and Mestre (2012) measured JNDs in path curvature, finding a Weber fraction of approximately 11 %. Bigler (2013) used the results of Legge and Campbell (1981), who found the angular resolution of the retina to be around 1.5 arc min, to calculate additive and multiplicative noise variances for visual perception of road path geometry ahead of the vehicle. However, these results were found by asking subjects to indicate when they could detect a change in position of a small dot, which is likely to be significantly easier than picking out the full road geometry from a complicated visual scene.

#### Otolith thresholds

Perception thresholds have been measured extensively for translational accelerations in the horizontal plane. Measurements have been carried out in the longitudinal (X) and lateral (Y) directions, and the thresholds have been seen to be similar in both directions (Benson et al. 1986); therefore, they are considered together. Thresholds have also been measured in the vertical (Z) direction (Nesti et al. 2014a); however, this is not so relevant for the car driver’s control task.

The ‘up–down’ method (Levitt 1970) was used to measure thresholds in several studies, with participants being subjected to sinusoidal stimuli with amplitudes which changed for each trial (Benson et al. 1986; Kingma 2005; Soyka et al. 2009, 2011; Hosman and Van Der Vaart 1978; Heerspink et al. 2005). Other studies used gradually increasing or decreasing motion amplitudes and asked subjects to indicate when they started or stopped perceiving motion (Hosman and Van Der Vaart 1978; Heerspink et al. 2005). The thresholds for decreasing amplitudes were found to be lower than the thresholds for increasing amplitudes. It was thought that this was because the subjects were already ‘tuned in’ to the signal so were able to pick it out from the noise more easily. In all of these studies, the subjects were moved in only one axis at a time while seated in the dark, so they were focused on the acceleration stimulus without any other distractions.

**Fig. 11:**
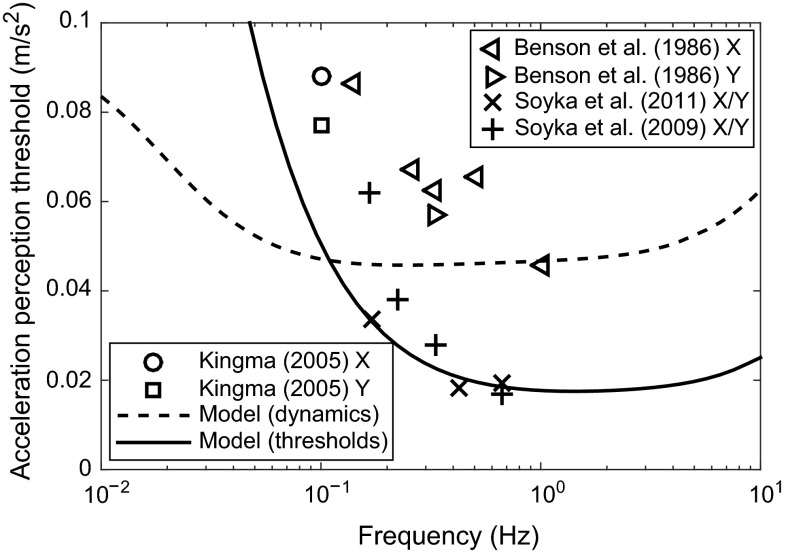
Lateral (Y) and longitudinal (X) acceleration thresholds measured in several different studies, compared with models found from the dynamics of the otoliths and from threshold measurements

Thresholds for the discrimination of the direction of sinusoidal accelerations in the horizontal plane from the studies using the up–down method are shown in Fig. 11. It is clear that there is a large variability in results between different studies and even within each study, indicating that perception thresholds are sensitive to differences in experimental methods and participants.

Predicted thresholds are also shown in Fig. 11, found using the signal-in-noise model of Soyka et al. (2011). The transfer function given in Eq. 2 was used with two different sets of parameters from Table 1. The dotted line shows the results using parameters found by Telban and Cardullo (2005) from the dynamics of the otoliths and measurements of brain responses, and the solid line shows the results using parameters optimised by Soyka et al. (2011) to fit the measured thresholds. The threshold model was found using the results of Soyka et al. (2011) only, whereas the noise level for the ‘dynamics’ transfer function was optimised to fit the whole data set. The noise levels found were 0.038 m/s$^2$* for the ‘dynamics’ transfer function and 0.015 m/s$^2$* for the ‘thresholds’ transfer function. The transfer function found from the threshold measurements fits the results much better than the transfer function found from the sensory dynamics, as the corner frequency at which the thresholds plateau is too low for the ‘dynamics’ transfer function.

Naseri and Grant (2012) measured JND values for sinusoidal accelerations at 0.4 and 0.6 Hz with varying amplitudes. The results were found to fit Weber’s law well, although a dependence on frequency was also seen. A Weber fraction of 5 % was found for the measurements taken at 0.4 Hz, whereas a value of 2 % was found for the measurements taken 0.6 Hz.

In interpreting the results of experiments which measure thresholds of whole body motion, the possibility of multimodal stimuli should be considered. For example, in the case of sinusoidal angular velocity imposed on the test subject, the semicircular canals and various somatosensors may be stimulated simultaneously. Multimodal thresholds and sensory integration are discussed in Sects. 4.3 and 5.

#### Semicircular canal thresholds

**Fig. 12:**
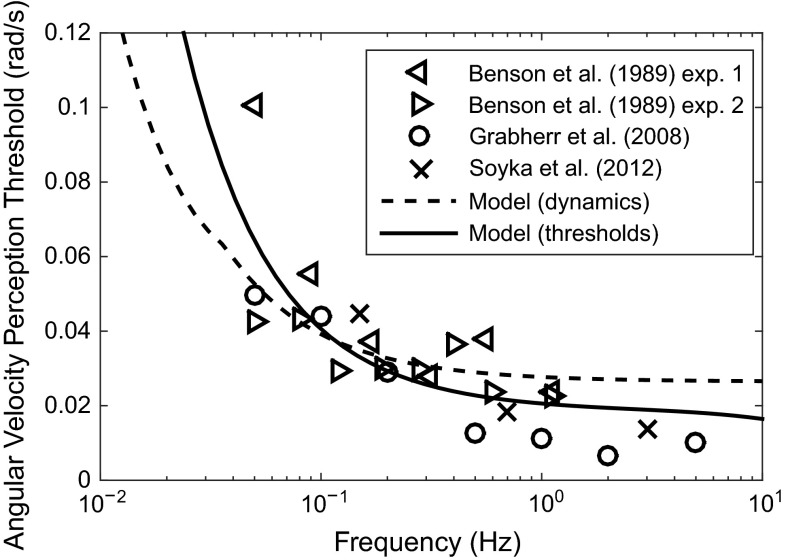
Yaw angular velocity thresholds measured in several different studies, compared with models found from the dynamics of the SCCs and from threshold measurements

Various studies have measured thresholds for perception of angular velocity, using either the up–down method (Benson et al. 1989; Grabherr et al. 2008; Soyka et al. 2012) or by gradually increasing or decreasing amplitudes (Hosman and Van Der Vaart 1978; Heerspink et al. 2005), in a similar way to the otolith measurements. Measured thresholds from studies using the up–down method are shown in Fig. 12. The data all follow a similar trend, with a fairly low amount of scatter compared to the otolith results. Predicted thresholds are also shown using the signal-in-noise model of Soyka et al. (2012), based on the transfer function given in Eq. 3. The solid line was found using parameters optimised by Soyka et al. (2012) to fit the threshold measurements, and the dotted line was found using the parameters suggested by Telban and Cardullo (2005) for the SCCs, choosing the noise level to fit the measured threshold parameters as well as possible. Both sets of SCC parameters are given in Table 2. The noise levels found were 0.025 rad/s* for the ‘thresholds’ transfer function and 0.023 rad/s* for the ‘dynamics’ transfer function. Both models fit the results well, although the model which was optimised to fit the threshold results matches more closely as expected.

JNDs for angular velocity perception have been measured by Mallery et al. (2010) and dos Santos Buinhas et al. (2013), finding Weber fractions of 3 and 13 %, respectively. The difference between these values may be a result of the fact that Mallery et al. (2010) measured JNDs at larger amplitudes than dos Santos Buinhas et al. (2013). Mallery et al. (2010) also found that the gradient (JND/amplitude) was higher at low amplitudes, and suggested a power law should be used rather than Weber’s law. However, it is debatable whether JNDs for the SCCs should follow a power law, when most other sensory systems have been found to follow Weber’s law.

#### Somatosensor thresholds

**Fig. 13:**
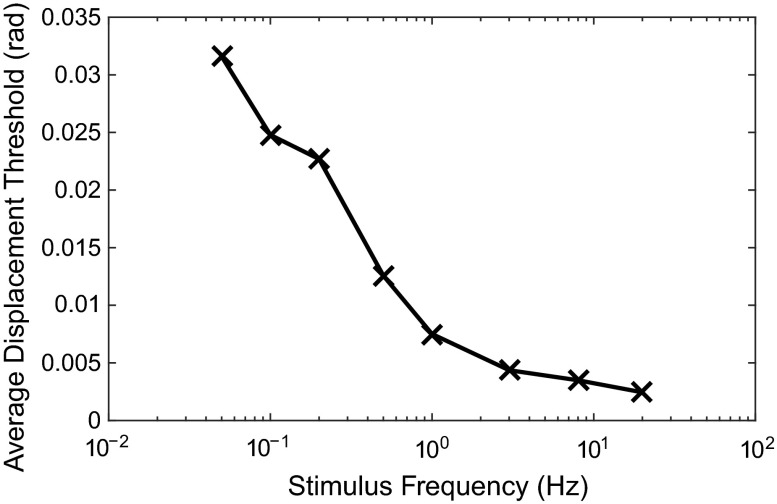
Thresholds for the perception of steering wheel angular displacement, measured by Bigler (2013). These results could be used to infer somatosensor noise levels; however, assumptions would have to be made about the integration of signals from the two muscle spindle afferents and the GTOs

Various studies have measured perception thresholds for the displacements of different limbs; however, Bigler (2013) is thought to be the first to directly measure thresholds for the perception of steering wheel angle, finding the results shown in Fig. 13. These results cannot be used to find noise levels for the somatosensors without making some assumptions about the relationship between steering wheel displacement and the displacements, velocities and forces of the muscles, and further assumptions about the method used to integrate information from the Ia, Ib and II afferents. Further work is necessary to determine appropriate noise levels for the somatosensors.

Newberry et al. (2007) measured JNDs in steering wheel angle and reported a Weber fraction of 14 %. However, this was achieved by fitting a line with zero perception threshold, and a better fit to the data can be achieved by including the effect of a nonzero perception threshold. This gives a good linear fit to the measurements, with a Weber fraction of 9.6 % and a perception threshold of 0.006 rad. The stimulus profile and frequency were not reported by Newberry et al. (2007); however, the extrapolated perception threshold is similar to that measured by Bigler (2013) for stimuli at 1 Hz (see Fig. 13).

To date, no studies appear to have directly measured perception thresholds for steering wheel force or torque. Steering wheel force JNDs were measured by Newberry et al. (2007), and extrapolating from these measurements gives a perception threshold of 0.45 N and a Weber fraction of 9.6 %. It is interesting to note that the Weber fraction found for the GTOs using this method matches the Weber fraction found for the muscle spindles almost exactly, suggesting that there may be a perceptual link between the two sensors. The GTO afferent and the primary muscle spindle afferent share the same nerve conduction path (Kandel et al. 2000), so since JNDs are related to noise along the transmission path this may provide an explanation for the similarity in Weber fraction values.

### Active and multimodal thresholds

The studies summarised in Sect. 4.2 were all targeted at measuring thresholds of a single stimulus in isolation, during passive conditions where the subject was concentrating on the stimulus. However, sensory stimuli which occur during driving are very different to the stimuli applied in these controlled studies, so these results may not be directly applicable to driving tasks. Stimuli in driving tasks are perceived under active rather than passive conditions, and there are several stimuli being perceived at once. Therefore, the indifference threshold [threshold in the presence of other stimuli (Groen et al. 2006)] should determine the limits of perception during driving.

By asking subjects to perform a secondary control task in a separate motion axis, it has been found that increasing the mental load on subjects causes an increase in perception thresholds (Hosman and Van Der Vaart 1978; Samji and Reid 1992). It should be noted that in both of these studies the subjects were still actively concentrating on perceiving the motion cues as well as completing the secondary task. Due to the equivalence of translational accelerations and shifts in the gravity vector, the brain can easily be fooled into misinterpreting the two types of motion. Groen and Bles (2004) and Pretto et al. (2014) found that presenting subjects with visual cues simulating a translational acceleration while they were undergoing rotational motion caused threshold of perception of the rotation to increase by factors of 5–6. Pretto et al. (2014) also measured thresholds during an active control task and found that they increased by factors up to 4 for some subjects, but didn’t change at all for others. The participants whose thresholds did not increase during the active driving task reported higher levels of ‘immersion’ in the simulation, indicating that the sense of realism of the simulation was linked to participants’ ability to perceive the motion cues accurately.

Pitch and roll thresholds have been measured with masking vertical motion cues, finding a significant linear increase in pitch and roll thresholds with vertical amplitude (Zaichik et al. 1999; Rodchenko et al. 2000). In contrast to these studies, Valente et al. (2006) found no significant effect of vertical motion amplitude on pitch rate thresholds. In this study, the pitch and vertical motion were applied at the same frequency, which may have caused the motion cues to be perceived as coherent, making it easier to detect the pitch cues.

Groen et al. (2006) analysed the data of Groen and Bles (2004) and showed that indifference thresholds for pitch rotation in the presence of visual longitudinal cues follow the same frequency response as the perception thresholds measured in passive conditions, but are increased by a constant gain. They used this result to hypothesise that the presence of additional sensory stimuli scales perception thresholds by a constant gain, without affecting the frequency response. This is consistent with the models of Soyka et al. (2011, 2012) and Bigler (2013) (shown in Fig. 9), where the threshold is placed after the sensory transfer function and the additional stimuli cause an increase in the noise level. Groen et al. (2006) suggested that the increase in noise level is linearly dependent on the amplitude of the additional stimulus, which is equivalent to Weber’s Law in the special case of the additional stimulus being in the same axis and modality as the measured stimulus.

**Fig. 14:**
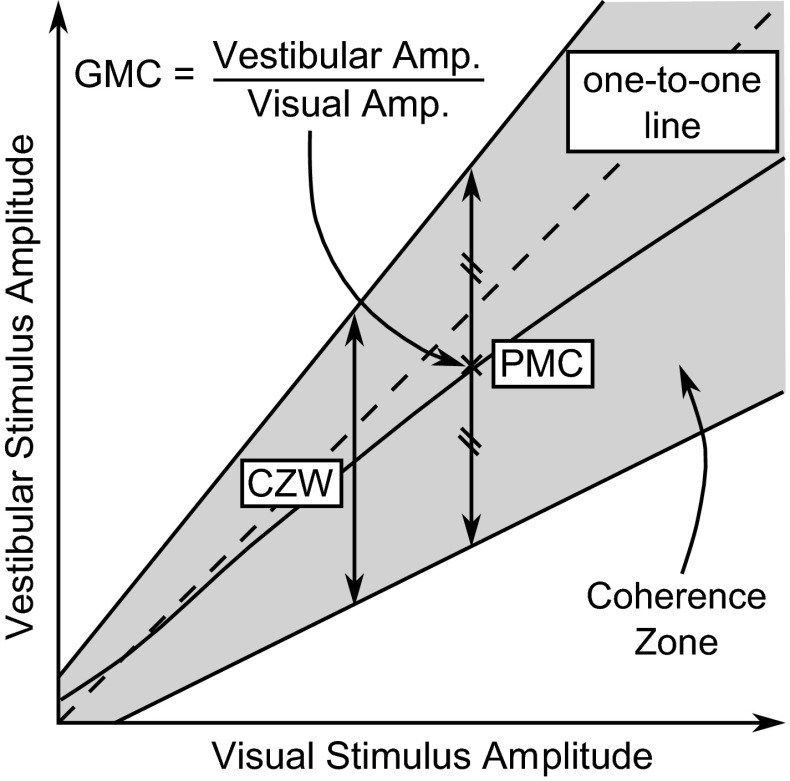
Coherence zone between visual and vestibular stimuli. For a given visual stimulus, there will be an upper and lower limit of vestibular stimulus amplitude which is perceived as coherent with the visual stimulus. The coherence zone width (CZW) is the difference between these two limits, and the point of mean coherence (PMC) is defined as the point halfway between the limits. The gain of mean coherence (GMC) is defined as the ratio of the vestibular amplitude to the visual amplitude at the PMC and represents the preferred gain between the visual and vestibular cues

Recent studies have used parameter identification methods to estimate threshold values during an active control task in the same axis, and thresholds in active conditions have been found to be around 1.6 times larger than thresholds measured in passive conditions (Pool et al. 2012; Valente Pais et al. 2012).

It is evident from the literature that various factors can cause thresholds to increase from values measured in passive conditions, including mental load, the presence of other stimuli and carrying out an active control task. It may therefore not be appropriate to rely on passive threshold measurements to model sensory dynamics during an active driving task.

### Coherence zones

The term ‘coherence zone’ was coined by van der Steen (1998) to describe the range of amplitudes of inputs to two sensory systems (such as visual and vestibular systems) which are perceived as consistent with each other, as shown in Fig. 14. The coherence zone can be defined in terms of the point of mean coherence (PMC), coherence zone width (CZW) and gain of mean coherence (GMC) as shown.

Coherence zones between the visual and vestibular systems have been measured at various amplitudes and frequencies (van der Steen 1998; Valente Pais et al. 2010a, b). The GMC was found to decrease with increasing stimulus amplitude, with subjects preferring larger vestibular motion than visual motion at low amplitudes and the opposite being seen at larger amplitudes. Significant differences were found between the values measured in different studies, highlighting the fact that coherence zones are highly dependent on the experimental conditions. Contrary to the results found for perception thresholds, coherence zones were found not to change significantly during an active control task (Valente et al. 2011). This indicates that the perceptual mechanisms behind perception thresholds and coherence zones may not be directly linked, and suggests that coherence zones measured in passive conditions may be applied to conditions where active control tasks are being carried out.

The concept of a coherence zone has been extended to the detection of heading direction (de Winkel et al. 2010) and phase differences (Grant and Lee 2007; Jonik et al. 2011). Jonik et al. (2011) found that inertial motion can lead visual motion by up to 22$^\circ$ without the difference being detected. This result was independent of the stimulus frequency, showing that humans can be considered as phase-error detectors rather than time delay detectors.

Research has shown that, when asked to tune inertial motion to match visual motion, subjects pick higher amplitudes when tuning downwards from high amplitude motion than when tuning upwards from low amplitude motion (Correia et al. 2010). Correia Grácio et al. (2013) defined the ‘optimal zone’ as the area between these ‘upper’ and ‘lower’ optima and found that it lay within the coherence zone. Similar to the PMC, GMC and CZW for coherence zones, the optimal zone was defined in terms of the ‘point of mean optimal gain’ (PMO), ‘gain of mean optimal’ (GMO) and ‘optimal zone width’ (OZW). The GMO was found to decrease at higher amplitudes and at higher frequencies. In contrast to coherence zone measurements, the OZW was found not to vary with amplitude or frequency. By varying the field of view, resolution and depth of the visual scene, Correia Grácio et al. (2014) found that the optimal gain is strongly affected by the ‘quality’ of the visual cues, with more realistic visual scenes giving GMOs closer to 1.

Two approaches to modelling CZWs were compared by dos Santos Buinhas et al. (2013), one matching the perceived intensity of the two stimuli and applying this to averaged JNDs, and one summing the JNDs for the two individual stimuli. Comparison of model predictions with experimental data showed that summing JNDs provides the best fit to the measured data, explaining the results particularly well at lower amplitudes. dos Santos Buinhas et al. (2013) suggested that PMCs could be modelled using Stevens’ power functions of perceived stimulus intensity; however, this method was not experimentally verified.

## Sensory integration

The sensory systems described in Sect. 2 provide the central nervous system (CNS) with measurements (or sensory ‘cues’) which can be used to estimate vehicle states while driving. However, these measurements are shaped by the sensor dynamics and also contain additive and signal-dependent noise (as described in Sect. 4). The CNS must therefore carry out sensory integration to give a single estimate of the vehicle states from the noisy, filtered information received from each of the sensors.

In a real-world driving scenario, the driver will be presented with coherent sensory information. Any discrepancies between information from the different sensors are due to sensory noise, or incomplete information available to a particular sensor. However, in some situations the information presented to the different senses may be incoherent or biased, in which case the driver may use a different integration strategy. This is particularly relevant for motion in virtual environments, where the visual, vestibular and somatosensory information presented to the driver may not all accurately reflect the real-world stimuli. An overview of methods and results from investigations of sensory integration in a variety of virtual environments (not specific to driving) is given by Campos and Bülthoff (2012). The following subsections build on this, focusing in more depth on results which suggest how information from the sensory systems summarised in Sect. 2 may be integrated during driving.

### Integration of coherent sensory measurements

The simplest model of sensory integration is a linear weighting of the estimates from different sensory systems (Hosman and Stassen 1999). Appropriate weightings can be found using sensory experiments; however, the scope of models with fixed weightings is likely to be limited. For many sensory systems, the CNS has been found to integrate measurements using statistically optimal methods (Ernst and Banks 2002; Oruç et al. 2003; Butler et al. 2010; Seilheimer et al. 2014). These methods are based on Bayes’ theorem (Bayes 1763), which relates the *a posteriori* probability $P(\hat{I}|\hat{S})$ of condition $\hat{I}$ given observation $\hat{S}$ to the probability $P(\hat{I}|\hat{S})$ of observation $\hat{S}$ given condition $\hat{I}$, the *a priori* probability $P(\hat{I})$, and the observation probability $P(\hat{S})$ (which is usually assumed uniform):

8 $$\begin{aligned} P(\hat{I}|\hat{S})= \dfrac{P(\hat{S}|\hat{I})P(\hat{I})}{P(\hat{S})} \end{aligned}$$

Optimal integration of sensory cues involves choosing from the set of all possible conditions $\hat{I}{=}\{\hat{I}_i|i{=}1,\ldots ,N_{\hat{I}}\}$ the condition $\hat{I}_i$ which has the highest probability $P(\hat{I}_i|\hat{S})$ based on the set of observations $\hat{S}= \{\hat{S}_i|i=1,\ldots ,N_{\hat{S}}\}$ from the different sensory channels. For a continuous set of possible conditions $\hat{I}$ a probability density function of $P(\hat{I}|\hat{S})$ can be plotted. Equation 8 shows that $P(\hat{I}_i|\hat{S})$ depends on an assumption about the probability distribution $P(\hat{I})$ before the measurements are made, known as a ‘prior’.

There are various ways in which the optimal value of $\hat{I}$ can be chosen, such as the ‘maximum a posteriori’ (MAP) estimate, the ‘minimum mean square error estimate’ (MMSE) and the ‘maximum likelihood estimate’ (MLE) (Clark and Yuille 1990; Vaseghi 2005). However, if the priors $P(\hat{I})$ and $P(\hat{S})$ are uniform and the probability distributions are symmetric, these estimates will be identical and can found by maximising the ‘likelihood’ function $P(\hat{S}|\hat{I})$.

If the probability distributions of the sensory estimates $\hat{S}_i$ are all Gaussian, the MLE $\hat{S}$ of a property is found by weighting each estimate in proportion to the inverse of its variance $\sigma _i^2$ (Yuille and Bülthoff 1996):

9 $$\begin{aligned} \hat{S} = \sum _{i} w_i \hat{S}_i \quad {\text {with}} \quad w_i = \dfrac{1/\sigma _i^2}{\sum _{j} 1/\sigma _j^2} \end{aligned}$$

The variance $\sigma ^2$ of the combined estimate $\hat{S}$ is found from Eq. 10 to be lower than the variances of the individual estimates from the different sensory systems:

10 $$\begin{aligned} \sigma ^2 = \left( \sum _{i} \dfrac{1}{\sigma _i^2}\right) ^{-1} \end{aligned}$$

Oruç et al. (2003) showed that a Gaussian prior can be included in the MLE analysis as an additional input, weighted by the inverse of its variance as usual. MacNeilage et al. (2007) used this result to model the integration of visual and inertial cues to disambiguate between an acceleration and a shift in the gravity vector, incorporating priors to model the assumptions that humans are normally in an upright position and that smaller accelerations are more likely than larger ones. Soyka et al. (2015) measured off-centre yaw rotation thresholds and found that SCC and otolith signals were integrated, although the results suggested information from additional sensory systems may also have been used.

Near-optimal Bayesian integration of visual and vestibular information has been measured in several studies (Gu et al. 2008; Butler et al. 2010; Fetsch et al. 2009; Prsa et al. 2012; Drugowitsch et al. 2014; Fetsch et al. 2010). In contrast to these studies, de Winkel et al. (2013) only found results that fit the MLE model for 3 out of 8 participants and Nesti et al. (2015) found that combined visual-inertial thresholds were higher than predicted by a MLE model. Butler et al. (2011) found that participants exhibited optimal visual–vestibular integration 90 % of the time with a stereoscopic visual display compared with 60 % of the time with a binocular display. This suggests that the realism of the visual scene may affect whether or not visual and vestibular information is integrated optimally. Some studies have found a slight over-weighting of one sense with respect to the other, although some of these have found that vestibular cues are weighted higher (Fetsch et al. 2009; Butler et al. 2010) while others have found that visual cues are weighted higher (Prsa et al. 2012). Prsa et al. (2012) suggested that over-weighting of otolith signals and under-weighting of SCC signals may occur when vestibular cues are integrated with visual cues.

In order to develop effective and efficient control strategies for interacting with their surroundings, humans use their experience to develop internal models of themselves and the world around them (Wolpert and Ghahramani 2000). They are able to use learning methods to adapt these models to changes in the environment (Wolpert et al. 2011) such as astronauts entering microgravity (Carriot et al. 2015). Using an internal model, a recursive state estimator can be used to provide new *a priori* estimates at each time step to give improved estimates of the system states. A common implementation of this method is the Kalman filter (Kalman 1960; Grewal and Andrews 2001). It is assumed that the observer has an internal model of the system given in state-space form:

11 $$\begin{aligned} \begin{aligned} {{\varvec{x}}}(k+1)&= {\mathbf {A}}{{\varvec{x}}}(k)+{\mathbf {B}}{{\varvec{u}}}(k)+{{\varvec{w}}}(k) \\ {{\varvec{y}}}(k)&= {\mathbf {C}}{{\varvec{x}}}(k)+{{\varvec{v}}}(k) \end{aligned} \end{aligned}$$

The main difference between a driver and a passenger is that the driver has perfect knowledge of the inputs ${{\varvec{u}}}$; however, they are perturbed by process noise ${{\varvec{w}}}$. Both driver and passenger measure the outputs ${{\varvec{y}}}$, which are perturbed by measurement noise ${{\varvec{v}}}$. The new estimate of the states $\hat{{{\varvec{x}}}}(k+1)$ is predicted by propagating the current input ${{\varvec{u}}}(k)$ and state estimate $\hat{{{\varvec{x}}}}(k)$ through the internal model of the system. A correction is then added based on the error between the previous estimated output ${\mathbf {C}}\hat{{{\varvec{x}}}}(k)$ and measured output ${{\varvec{y}}}(k)$, weighted by the ‘Kalman gain’ ${\mathbf {K}}(k)$:

12 $$\begin{aligned} \hat{{{\varvec{x}}}}(k+1) = {\mathbf {A}}\hat{{{\varvec{x}}}}(k)+{\mathbf {B}}{{\varvec{u}}}(k)+{\mathbf {K}}(k)\left\{ {{\varvec{y}}}(k)-{\mathbf {C}}\hat{{{\varvec{x}}}}(k)\right\} \end{aligned}$$

The time-varying Kalman gain ${\mathbf {K}}(k)$ is calculated to give a statistically optimal estimate using MLE, weighting the estimates based on the covariances of the Gaussian noise ${{\varvec{w}}}$ and ${{\varvec{v}}}$. If the covariances are time invariant, a steady-state linear filter can be found to give the optimal state estimates for the system. Various studies have proposed models of visual–vestibular integration based on Kalman filters (Borah et al. 1988; Zupan et al. 2002; Young 2011), and Kalman filters have also been used to model estimation of vehicle states for pilots (Onur 2014) and drivers (Bigler 2013).

**Fig. 15:**
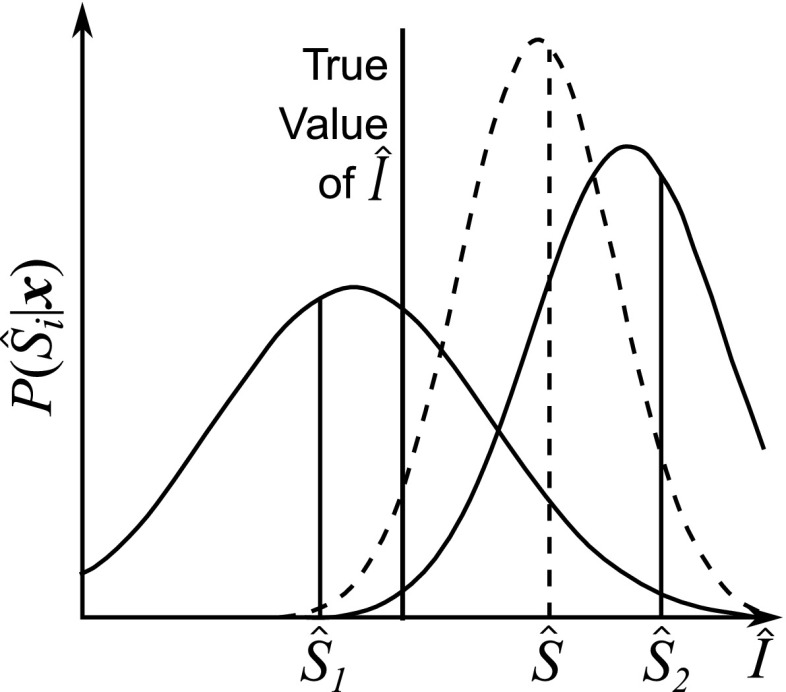
Maximum likelihood integration of two biased sensory channels. The probability distributions of the sensors $\hat{S}_1$ and $\hat{S}_2$ (shown by the *solid lines*) are both biased, i.e. their means do not correspond to the true value of $\hat{I}$. Using MLE causes this bias to carry through to the combined estimate given by the *dashed line*. Also note that the sensor with the largest variance does not necessary have the largest bias

One implication of MLE models of human sensory integration is that the observer must have access to estimates of the noise variance for each sensory channel. Ernst and Bülthoff (2004) suggested that the variance may be determined by looking at the responses over a population of independent neurons. Several studies have attempted to build realistic neural models to describe this behaviour (Deneve et al. 1999; Pouget et al. 2000; Barber et al. 2003), and they have found that a close approximation to MLE can be achieved in such a way. Fetsch et al. (2009) studied the integration of visual and vestibular cues to heading angle in humans and monkeys with varying reliability of the visual cues. They found that both humans and monkeys were able to dynamically re-weight the cues between trials, indicating that they were able to obtain a measure of the reliability of each cue.

### Integration of biased sensory measurements

While MLE is an optimal method of combining measurements from noisy sensory channels with the same mean, if the signals are biased such that their means are no longer coherent, using MLE will cause the bias to carry through into the ‘optimal’ sensory estimate as seen in Fig. 15 (Ernst and Luca 2011). There will always be differences between the measurements $\hat{S}_i$ from different noisy sensory channels; however, it is impossible to separate these differences into those which occur because of stochastic variations about the mean and those which are a result of biases in the sensory channels without prior knowledge of the values of these biases. It has been found that the CNS may ignore the discrepancies and integrate the biased sensory measurements using the MLE method if conflicts are small (Scarfe and Hibbard 2011; Butler et al. 2010, 2014) or if the conflicting information is presented in different motion axes (Kaliuzhna et al. 2015). de Winkel et al. (2015) found that over half of subjects integrated visual and inertial heading information regardless of the size of the bias. However, other studies have found evidence of various strategies for reducing bias in perceived signals (Körding et al. 2007; Landy et al. 1995; Burge et al. 2010; Zaidel et al. 2011).

When presented with two different sensory cues, the CNS must decide whether or not they are coherent (originating from the same source). If they are coherent, the difference between them can be assumed to be a result of stochastic variations and the cues can be combined using MLE. If not, the cues should be treated separately, treating the situation as a ‘cue conflict’. Körding et al. (2007) proposed a model using Bayes’ rule to decide whether or not two cues are coherent based on a prior describing the likelihood of the cues coming from the same source. They validated the model using experimental results; however, Seilheimer et al. (2014) noted that Körding et al. (2007) did not vary the reliability of the cues, so it is still uncertain whether their model is valid in all cases. A similar Bayesian model incorporating priors was proposed by Knill (2007). They found that the weight applied to a cue shrunk as the size of the conflict increased, but it did not decrease to zero. However, other studies have found that under some circumstances humans will ‘veto’ a cue that does not fit with the other sensory measurements (Girshick and Banks 2009; Landy et al. 1995).

Ghahramani et al. (1997) proposed an additional stage of ‘cue calibration’ before cues are fully integrated, where the difference between the estimates is reduced. The values of estimates $\hat{S}_1$ and $\hat{S}_2$ are calibrated by adding $\delta \hat{S}_1$ and $\delta \hat{S}_2$, given by:

13 $$\begin{aligned} \begin{aligned} \delta \hat{S}_1&= C_1 \times (\hat{S}_2-\hat{S}_1) \\ \delta \hat{S}_2&= C_2 \times (\hat{S}_1-\hat{S}_2) \end{aligned} \end{aligned}$$

This improves ‘internal consistency’ (Burge et al. 2010), ensuring that the estimates from different sensory systems agree with each other, although it does not necessarily improve ‘external accuracy’ (the overall accuracy of the CNS’s combined estimate). If $C_1+C_2=1$, calibration can achieve full internal consistency by adjusting both estimates to the same value; otherwise, a smaller reduction in the difference between the estimates is found.

Several studies have shown that vision dominates the other senses under certain conditions (Rock and Victor 1964; Ernst and Banks 2002), so a model of ‘visual capture’ has been proposed where vision completely dominates the combined estimate (Ernst and Banks 2002; Ernst and Bülthoff 2004). In such a case, the visual estimate does not change and the other estimate adapts to be the same as the visual estimate, giving $C_i=0$ for the visual channel and $C_i=1$ for the other channel. Alternatively, Ghahramani et al. (1997) proposed that the calibration stage follows a similar weighting structure to the integration process, based on reliability. He hypothesised that each calibration constant $C_1$ is proportional to the variance $\sigma _i^2$ of the sensory estimate $\hat{S}_i$. Burge et al. (2010) tested this model in an experiment on visual-haptic estimation of slant and reported strong evidence in favour of this reliability-based calibration.

Reliability-based calibration does not make physical sense as a method for reducing sensory bias, however, as the reliability of a cue is independent of its bias (Ernst and Luca 2011). A sensory estimate could have low variance (high reliability) and high bias, or a high variance (low reliability) but low bias. For example, in Fig. 15, the cue with the higher variance has the lower bias. Testing a ‘cue veto’ model of integration of biased sensory estimates, Girshick and Banks (2009) found that the vetoed cue was not necessarily the cue with the highest variance. Zaidel et al. (2011) compared reliability-based calibration with fixed-ratio calibration, where the calibration constants $C_i$ were assumed to be learned from past experience. Fixed-ratio calibration fitted their results better than reliability-based calibration, with higher weighting placed on the visual estimate. The sum of the calibration constants $C_1$ and $C_2$ was found to be less than 1, so full internal consistency was not achieved. Zaidel et al. (2011) also explained how the presence of fixed-ratio calibration could cause erroneous indications of reliability-based calibration to appear using the methods of Burge et al. (2010). It therefore seems that with biased sensory information humans may use fixed calibration constants based on past experience, rather than change the weightings based on cue reliability.

Linear cue calibration for visual–vestibular integration has been observed in several studies although, as with coherent measurements, there is disagreement about which sense is more highly weighted. Visual dominance was found by Rader et al. (2011), whereas the vestibular system was found to dominate by Harris et al. (2000). Ohmi (1996) found that visual cues dominated when conflicts were small, but vestibular cues dominated when conflicts were large. Zacharias and Young (1981) found that vestibular cues dominated visual cues at higher frequencies, so the dominant sensory system may depend on the frequency content of the tasks carried out.

**Fig. 16:**
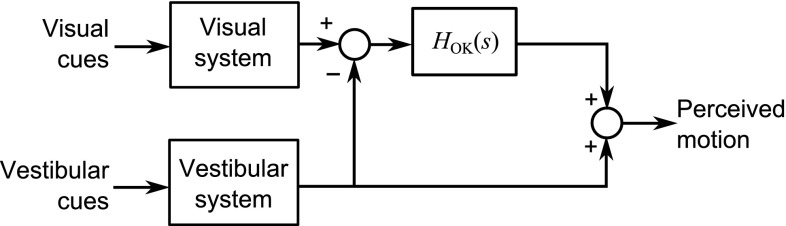
Model of the ‘optokinetic influence’ proposed by van der Steen (1998). The difference between the visual and vestibular estimates is passed through a low-pass filter $H_{\mathrm{OK}}(s)$ and then added to the vestibular estimate

**Fig. 17:**
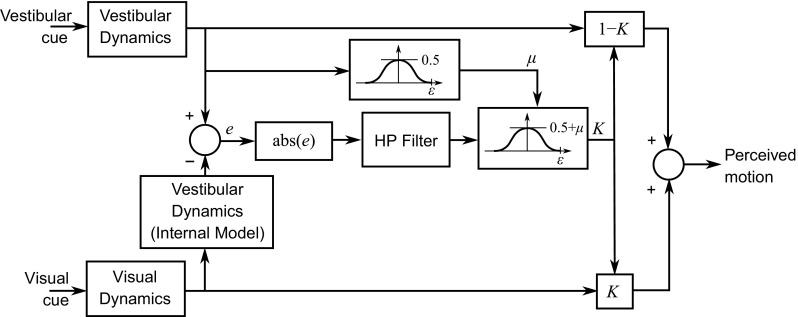
Visual–vestibular integration model proposed by Zacharias (1977). Figure adapted from Telban and Cardullo (2005). The visually perceived motion is filtered through an internal model of the vestibular dynamics, producing an ‘expected’ vestibular signal which is subtracted from the actual vestibular signal to give an error term. In order to allow resolution of steady-state cue conflicts, the error is washed out using a high-pass filter. The weighting on the visual and vestibular cues depends on the gain *K*, which varies between 0 and 1 based on symmetric weighting functions, chosen as cosine bell functions for simplicity. A large conflict will drive *K* to zero, vetoing the visual cue [matching the vestibular dominance seen for large conflicts (Ohmi 1996)]. A small conflict will cause *K* to be between 0.5 and 1, depending on the magnitude of the visual cue. This behaviour was chosen to qualitatively match the transient perception of motion observed in cue-conflict situations

Experimental studies have shown that when a consistent conflict is observed between the visual and vestibular systems, the perceived motion will eventually drift towards the visual estimate (Ishida et al. 2008). van der Steen (1998) proposed a model of the ‘optokinetic influence’, where the visual estimate ‘attracts’ the vestibular estimate over a transient period, modelling the onset of visual self-motion (‘vection’). This is modelled by passing the difference between the visual and vestibular estimates through a low-pass filter given by:

14 $$\begin{aligned} H_{\mathrm{OK}}(s) = \frac{1}{1+\omega _{\mathrm{OK}}s} \end{aligned}$$

giving the optokinetic influence which is then added to the vestibular output as shown in Fig. 16. An implication of this model is that pre-filtering the vestibular cues by the inverse of the vestibular dynamics, or conversely pre-filtering the visual cues by the vestibular dynamics, should cause the visual and vestibular cues to be perceived as coherent even though they differ substantially. Wentink et al. (2009) tested this hypothesis using subjective feedback from experiments in a simulator and found that pre-filtering the vestibular cues by the inverse of the vestibular dynamics did indeed result in coherent perception. However, pre-filtering the visual cues by the vestibular dynamics produced cues which were perceived as coherent for only half the motion conditions.

**Table 3:** Summary of sensory system results from the literature

System	Input	From sensory dynamics		From perception thresholds		Weber fraction (%)	Sensor delay (ms)
		Transfer function	Noise	Transfer function	Noise		
Visual feedback	Yaw angular velocity	1	0.0013 (rad/s*)	$\dfrac{0.810}{s + 0.810}$	0.0011 (rad/s*)	7–11	100–560
Visual feedback	Lateral velocity	1	0.035 (m/s*)	$\dfrac{0.810}{s + 0.810}$	0.032 (m/s*)	7–11	100–560
Visual feedback	Longitudinal velocity	1	–	–	–	10–50	100–560
Visual feedforward	Target path	Preview model	–	–	–	11	100–560
Otoliths	Acceleration	$\dfrac{0.4(1+10s)}{(1+5s)(1+0.016s)}$	0.038 (m/s$^2$*)	$\dfrac{0.0225(1+22.05s)}{(1+0.62s)(1+0.016s)}$	0.015 (m/s$^2$*)	2–5	5–440
SCCs	Angular velocity	$\dfrac{5.73(80s^2)}{(1+80s)(1+5.73s)}$	0.023 (rad/s*)	$\dfrac{2.2s(1+0.014s)}{(1+2.16s)(1+0.005s)}$	0.025 (rad/s*)	3–13	5–440
Muscle spindles (Type Ia)	Arm muscle displacement	$\dfrac{s(s+0.44)(s+11.3)(s+44)}{(s+0.04)(s+0.816)}$	–	–	–	10	>34
Muscle spindles (Type II)	Arm muscle displacement	$\dfrac{(s+0.44)(s+11.3)}{(s+0.816)}$	–	–	–	10	>48
GTOs	Arm muscle force	$\dfrac{333(s+0.15)(s+1.5)(s+16)}{(s+0.2)(s+2)(s+37)}$	–	–	–	10	>34

For the key sensory systems involved in driving, transfer functions between the input stimulus and the sensory response are given, either from considerations of the sensory dynamics or from perception threshold measurements. Noise levels have been calculated from sensory threshold measurements, as well as Weber fractions showing how thresholds increase with stimulus amplitude. Estimates of sensory delays are also included

Zacharias (1977) and Zacharias and Young (1981) developed a detailed empirical model of visual–vestibular integration under cue-conflict conditions, shown in Fig. 17. Borah et al. (1988) developed an adaptive version of the model of Zacharias (1977), using a slightly modified weighting function then multiplying the visual estimate by the gain *K* and combining it with the vestibular estimate using a Kalman filter. Telban and Cardullo (2005) adapted the model of Zacharias (1977), using some of the modifications suggested by Borah et al. (1988) and including the optokinetic influence modelled by van der Steen (1998). They ran simulations to find the response to velocity step inputs to the visual system with and without corresponding vestibular cues and found that it was possible to reproduce latencies measured in previous studies on humans. However, further validation work is needed to determine whether this model is more generally applicable.

Wright et al. (2005) subjected participants to vertical motion with conflicting visual and vestibular information, playing back recordings of the visual surroundings of the apparatus to give a realistic scene. Visual and vestibular cues were presented with different amplitudes and in some cases out of phase with one another. The results were found to be incompatible with linear weighting conflict models and the more complicated model of Zacharias (1977), as for high visual amplitudes the visual perception was found to dominate, independent of the vestibular amplitude. More research is clearly needed to develop a model which can describe integration of biased sensory estimates under a wide range of conditions.

## Discussion

Key results from the literature on human sensory dynamics have been presented in Sects. 2 to 5. In this section, these results are summarised and discussed with a view to understanding and modelling driver steering and speed control.

Results for the human sensory systems which are most relevant to driver modelling are summarised in Table 3. Transfer functions are presented which have either been found from models of the sensory dynamics and measurements of brain activity or inferred from sensory threshold measurements. Using the transfer functions found from sensory threshold data may give more accurate results near the limits of perception; however, they may not capture all of the dynamic behaviour of the sensory system.

Noise magnitudes have been inferred from sensory threshold measurements using the signal-in-noise model of Soyka et al. (2011, 2012). These were found from passive threshold measurements taken for one sensory stimulus at a time; however, thresholds have been found to increase in active conditions and in the presence of other sensory stimuli by factors between 1.5 and 6 (Hosman and Van Der Vaart 1978; Samji and Reid 1992; Zaichik et al. 1999; Rodchenko et al. 2000; Groen and Bles 2004; Valente Pais et al. 2012). This means that the noise values shown in Table 3 should be considered as lower bounds. Most sensory systems have been found to approximate Weber’s law, with JNDs increasing with stimulus amplitude; therefore, Weber fractions have been included in Table 3. This increase in sensory noise with stimulus amplitude can be modelled by including signal dependent as well as additive noise (Todorov 2005; Bigler 2013).

Estimates of sensor delays are also given in Table 3 for each system, comprising of all components of the time delay between stimulus application and physical response. However, there is still some uncertainty about the precise values, as it is thought that delays in neural processing may be dependent on the exact nature of the stimuli and the task being carried out. It is unclear whether delays increase or decrease during active conditions; however, they have been found to increase with additional stimuli in multimodal conditions.

For many types of stimuli, coherent sensory information has been found to be integrated in a statistically optimal fashion (Ernst and Banks 2002; Oruç et al. 2003; Butler et al. 2010, 2011; Seilheimer et al. 2014; Gu et al. 2008; Prsa et al. 2012; Drugowitsch et al. 2014). Humans build up internal models of themselves and their surroundings (Wolpert and Ghahramani 2000) and a Kalman filter can be used to model optimal sensory integration using internal models (Kalman 1960; Grewal and Andrews 2001; Borah et al. 1988; Zupan et al. 2002; Young 2011; Onur 2014). For incoherent sensory information, when conflicting information is presented to the different sensory channels, sensory integration is less well understood. A variety of different models have been proposed; however, no overwhelming evidence has been found in favour of any of them. Consideration of how humans integrate incoherent or biased sensory measurements may be important when studying drivers in virtual environments; however, in normal driving the senses should be in agreement.

Since sensory parameters have been found to change under active or multimodal conditions, it may not be appropriate to apply the results shown in Table 3 directly to a driver model. It is very difficult to measure sensory parameters directly during realistic driving conditions. However, by developing a model of driver control behaviour incorporating sensory dynamics, parametric identification methods could be used to gain some insight into the performance of sensory systems while driving. Parametric identification procedures have been described by Ljung (1999) and Zaal et al. (2009b) and applied to driver models by Keen and Cole (2012) and Odhams and Cole (2014). A number of studies have been carried out at Delft University of Technology to identify pilot control strategies under different conditions (Pool et al. 2008; Zaal et al. 2013, 2009c, 2010, 2009a, 2012; Nieuwenhuizen et al. 2013; Ellerbroek et al. 2008; Drop et al. 2013). Since the pilot control task is similar to a driver steering control task, the results of Zaal et al. (2009c) have been used to validate an initial concept for a driver model incorporating sensory dynamics (Nash and Cole 2015).

It is hoped that the information presented in this literature review will inform and motivate future researchers to consider the influence of sensory dynamics in driving tasks. The differences highlighted here between active and passive measurements mean that we do not advocate direct incorporation of results from the sensory perception literature into driver models. Rather, identification methods used in recent aerospace studies seem to show more promise, and there is clear scope for applying similar techniques to studying drivers. The danger in such an approach is that identification of a large number of sensory parameters may become infeasible. Therefore, care must be taken to increase the complexity of sensory models slowly and use carefully designed experiments to isolate different features of sensory perception during driving. This review should serve as a guide for potential areas of investigation and a reference to compare with new results.

## Conclusion

The results summarised in this literature review give an insight into various different sensory systems, and how they can be used to model driver control behaviour. Sensory transfer functions have been studied extensively, and there is little disagreement between different studies. Sensory integration is reasonably well understood under normal conditions; however, there is little agreement on how humans cope with conflicting sensory information. Studies have shown that sensory thresholds increase under active and multimodal conditions, but further research is necessary to determine how and why this happens. Time delays also increase during multimodal conditions; however, it is not clear whether they vary during active control tasks. There is a great deal of scope for improvement in the available knowledge on human sensory perception during active control tasks, so future research should focus in this area. It is hoped that the information in this review will prove useful in developing more sophisticated driver steering and speed control models which take account of the driver’s sensory dynamics.
